# An Unusual Case Report of COVID-19 Presenting with Meningitis Symptoms and Shingles

**DOI:** 10.5811/cpcem.2020.4.47557

**Published:** 2020-04-29

**Authors:** Rebecca Packwood, Gayle Galletta, Joseph Tennyson

**Affiliations:** University of Massachusetts, Department of Emergency Medicine, Worcester, Massachusetts

**Keywords:** COVID-19, novel coronavirus, meningitis, shingles, presenting symptoms

## Abstract

**Introduction:**

As severe acute respiratory syndrome coronavirus 2 (SARS-CoV-2) spreads across the globe, physicians face the challenges of a contagious pandemic including which patients to isolate, how to conserve personal protective equipment, and who to test. The current protocol at our hospital is to place anyone with new cough, dyspnea, or fever into airborne and contact precautions and consider them for testing. Unfortunately, the symptomatic presentations of coronavirus disease 2019 (COVID-19) are proving more variable than previously thought.

**Case Report:**

Our case of COVID-19 presented with headache and then progressed to a meningitis-like illness with co-existing shingles rash.

**Conclusion:**

COVID-19 can have a variety of initial presentations that are not the classic respiratory symptoms and fever. These presenting symptoms of COVID-19 can include a meningitis-like illness, as our case report indicates. The wide variety of presentations of COVID-19 may warrant widespread testing to identify cases, protect healthcare workers, and prevent the spread of this pandemic.

## INTRODUCTION

The year 2020 will forever be defined by the spread of severe acute respiratory syndrome coronavirus 2 (SARS-CoV-2, previously 2019-nCoV), which causes coronavirus disease 2019 (COVID-19). COVID-19 can cause a devastating bilateral, multilobar pneumonia, acute respiratory distress syndrome, and death. The first identified cases appeared in Wuhan, China, in December 2019. The World Health Organization declared COVID-19 a public health emergency in February 2020, and the United States (US) currently has the highest burden of cases of any country.^7^

One of the highest priorities for patients and healthcare workers is identifying the presenting symptoms of COVID-19. Studies from China indicate that fever, cough, and dyspnea are among the most common presentations of the disease. Huang et al published one of the first prospective case studies of 41 patients in Wuhan and found that the most common symptoms were fever (98%) and cough (76%).^3^ The complete range of clinical manifestations included fever, non-productive cough, dyspnea, myalgia, and fatigue.^3^

Here in the US, patients with “concerning symptoms” are placed in isolation. Our institution’s COVID-19 triage screening includes new cough, new shortness of breath, fever, recent travel, or known COVID-19 contacts. Initially, patients who screened positive for multiple risk factors were placed in a negative pressure room (if available) on airborne and contact precautions. However, as cases increased, the screening criteria expanded to patients who screened positive for any of the screening questions above. While these questions are consistent with the most common presenting symptoms, the range of clinical symptoms of this disease is varied and our screening questions are missing patients. This has clinical significance for both the patients, who have delayed diagnosis, as well as the healthcare providers who experience unprotected COVID-19 exposures.

## CASE REPORT

A 58-year-old-male with history of hyperlipidemia presented to the emergency department (ED) with the chief complaints of headache, abdominal pain, and constipation. The patient started having mid-to-lower abdominal discomfort associated with constipation two days prior to presentation. On the day of presentation, his discomfort worsened and he noted a fever of 100.7 degrees Fahrenheit (F). He also developed a progressively worsening headache located in bilateral occiputs and radiating to his neck. He denied a history of migraines. Patient denied international travel but had traveled to Florida the week prior. No cough, dyspnea, or known COVID-19 contacts were reported.

Vital signs revealed a temporal temperature of 36.6° Celsius (C), heart rate 93 beats per minute (bpm), respiratory rate 18 breaths per minute, blood pressure 130/83 millimeters of mercury (mmHg), and oxygen saturation (SpO_2_) 98% on room air. He had clear lung sounds bilaterally, a normal cardiovascular exam, and mild tenderness in the right upper quadrant. He was also tender at the bilateral inserts of the suboccipital muscles. The patient was neurologically intact with a Glasgow Coma Scale of 15, normal cranial nerves, and no motor or sensory deficits. He had full range of motion of his neck and no meningismus.

Laboratory results were remarkable for a normal white blood cell count of 5.3 × 10^3^ microliters per liter (uL) (reference range 4.3–10.8 × 10^3^/uL) but with lymphopenia of 0.4 × 10^3^/uL (reference range 0.9–3.4 × 10^3^/uL). Lactic acid, basic metabolic panel, hepatic panel, and lipase were within normal limits. Computed tomography (CT) of the abdomen/pelvis showed no acute abnormality but did note minimal bibasilar atelectasis.

The patient’s headache initially improved with intravenous (IV) fluids and metoclopramide but later recurred. Due to the location of his headache and tenderness at the suboccipital muscle inserts, a bilateral occipital nerve block with 0.5% bupivacaine was performed with improvement of his pain. Shared decision-making was conducted with the patient and a lumbar puncture (LP) was declined. He was discharged home with a diagnosis of non-specific viral syndrome and strict return precautions.

Three days after his initial presentation, the patient continued to have fevers, headaches, and developed a dermatomal rash. He was started on famciclovir for presumed shingles.

The patient re-presented to an affiliated ED six days after his initial presentation for fever, headache, neck pain, and diffuse abdominal pain. He also noted fatigue, myalgias, dyspnea, congestion, and rash. Vital signs revealed a temperature of 37.1° F, heart rate 94 bpm, respiration rate 16 breaths per minute, blood pressure 96/66 mmHg and SpO_2_ 98% on room air. The physical exam was now more concerning for meningitis with neck rigidity and pain with neck movement. A vesicular rash along the right ninth and tenth thoracic dermatomes was also noted. The patient was otherwise neurologically intact and his pulmonary exam was normal. Due to the presence of the shingles rash, he was placed on strict airborne and contact precautions for concern of disseminated herpes zoster infection.

**CPC-EM Capsule**

What do we already know about this clinical entity?

Severe acute respiratory syndrome coronavirus 2 (SARS-CoV2) which causes coronavirus disease 19 (COVID-19) appeared in Wuhan, China in December, 2019. It reportedly presents with shortness of breath, cough and fever.

What makes this presentation of disease reportable?

Our case report reviews a meningitis-like presentation of COVID-19 including symptoms of headache, meningismus and fever. Due to the unusual presentation, this patient had a delay in diagnosis.

What is the major learning point?

COVID-19 presents with a more varied array of symptoms then previously identified which places patients at risk for delayed diagnosis and caregivers at risk of exposure.

How might this improve emergency medicine practice?

As our testing capability expands, all patients presenting to the emergency department should be placed on precautions and tested in order to improve diagnosis and prevent caregiver exposures.

**Image 1:**
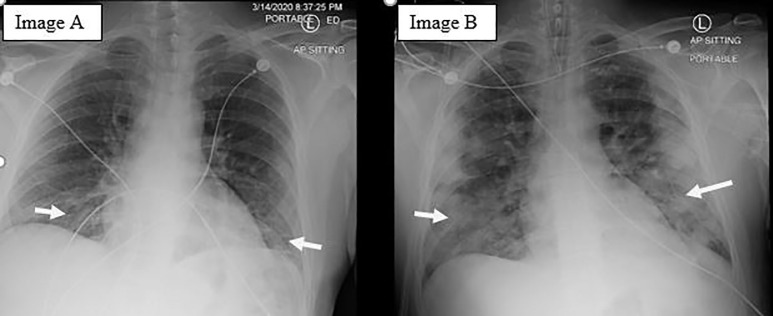
A) Portable chest radiograph on presentation to the emergency department, demonstrating subtle, patchy infiltrates visible in the lung bases suggesting early pneumonia (arrows). B) An anterior posterior chest radiograph two days after admission, demonstrating significant interval progression of peripherally located patchy opacities throughout both lungs (arrows) with areas of consolidation at the lung bases and right upper lobe.

An LP was performed due to concern for meningitis and the patient was started on IV acyclovir, vancomycin, and ceftriaxone. The LP revealed an opening pressure of 21 centimeters of water (cm H_2_O) (normal range 10–20 cm H_2_O), elevated glucose of 84 milligrams per deciliter (mg/dL) (normal range 45–80 mg/dL or greater than 60% of serum glucose), elevated protein of 48 mg/dL (normal less than 45 mg/dl), and one white blood cell (WBC) in both tubes one and four (normal range 0–5 cells/microliter). Infectious disease consult later remarked that there was a low suspicion for meningitis based on his LP. A chest radiograph (CXR) showed subtle, patchy infiltrates in the lung bases (Image 1) suggesting early pneumonia, so the patient’s antibiotics were expanded to include IV doxycycline to cover atypical bacteria.

**Image 2:**
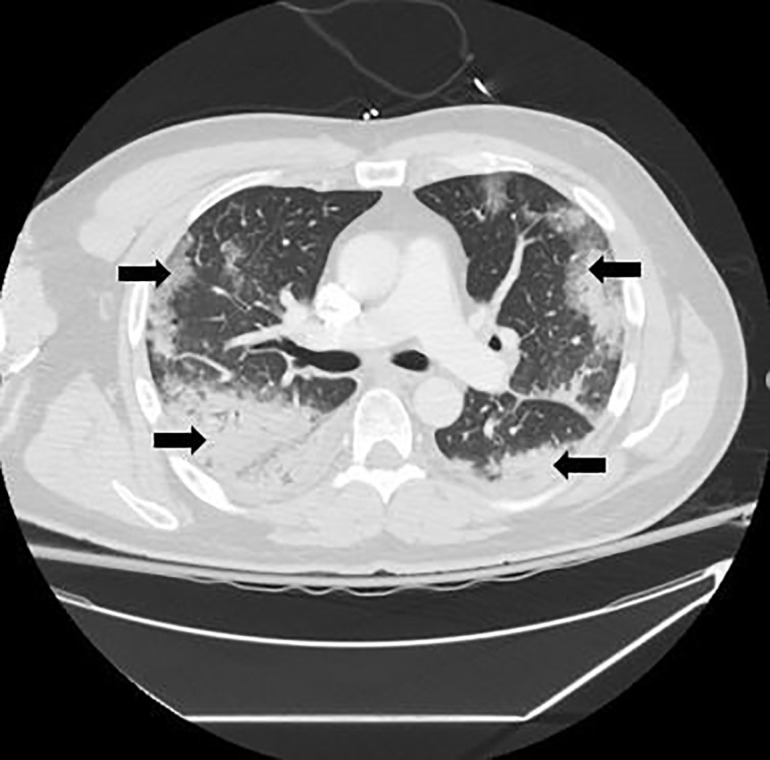
Computed tomography of the chest two days after admission demonstrating extensive, bilateral multifocal pneumonia most notable in the lower lobes (arrows).

The patient was admitted to the general medicine floor for observation pending cerebrospinal fluid culture results. On day seven after his initial presentation, precautions were reduced from strict airborne to droplet and contact precautions. He symptomatically improved until the early morning of day eight after his initial presentation, when he started to complain of acute left-sided, pleuritic chest pain and shortness of breath. A rapid response was called. The patient was treated for possible acute coronary syndrome and further evaluated with a CXR and CT-pulmonary embolism protocol. Both the CXR (Image 1) and CT (Image 2) showed a multilobar peripheral pneumonia, which was highly concerning for COVID-19 infection.

Eight days after his initial presentation (day two of hospitalization), the patient was placed on strict airborne, contact, and droplet precautions. His respiratory status continued to deteriorate and he was transferred to the intensive care unit (ICU) on high-flow nasal cannula and required intubation later that day. His COVID-19 test via the Department of Public Health was presumptive positive.

The patient was intubated in the ICU from day two of hospitalization until day 15. During his extensive ICU course, various treatments were trialed including lopinavir/ritonavir, a six-day course of hydroxycholorquine and azithromycin, as well as remdesivir starting hospital day 19. He was also continued on his course of antibiotics.

In a study from Wuhan the average time from onset of first symptoms to dyspnea was five days, to admission was seven days and to acute respiratory distress syndrome was eight days.^4^ This patient was admitted on day nine, decompensated on day 11 and was intubated for a total of 13 days. The patient’s clinical course is summarized in Table 1.

## DISCUSSION

This case draws to light the significant COVID-19 exposure risk to both the ED and medical floor staff. This patient had two ED visits as well as two days on the general medicine floor prior to initiation of full airborne and contact precautions due to his atypical symptoms on presentation.

A literature review revealed that while the most common presenting symptoms are fever and cough, there is a dramatic range of symptoms, which can be associated with COVID-19. In a single-center, retrospective study of 54 healthcare workers who succumbed to COVID-19 in Wuhan, fever was the most common presenting symptom followed by cough.^1^ Another retrospective study of 138 patients in Wuhan also confirmed that the most common presenting symptoms were fever, fatigue, and cough.^4^ However, both of these studies also showed significant prevalence of other symptoms such as myalgias, headache, nausea and diarrhea, which are typically not represented in our screening questions. Between 4–13% of patients presented with headache. Notably, no cases described a meningitis-like presentation as our patient specifically demonstrated. In addition, the *New England Journal of Medicine* published a meta-analysis of 1099 patients hospitalized across 552 sites as of January 29, 2020. In terms of clinical symptoms on presentation, only 43.8% of patients had fever on presentation but 88.7% developed fever during hospitalization. A fever was defined as axillary temperature of greater than 99.5° F (37.5° C). Cough was noted in 67.8% and headache in 13.6% of patients. See Table 2 for a full table reviewing symptom prevalence in each of these studies.

Interestingly, Deng et al compared the presenting symptoms of patients with COVID-19 who progressed to severe respiratory illness vs those who remained mildly symptomatic. This retrospective study of 225 patients found statistically significant differences in the presenting clinical symptoms of these patients.^5^ Patients who presented with dyspnea, expectoration, low oxygen saturations, and severe illness were more likely to progress to death.^5^ In addition, the average day of admission for the group who recovered was day seven while the average day of admission for the group who died was day 10.^5^ Patients who presented later in their clinical course appeared to progress to more severe illness.^5^ While our patient presented later and did progress to severe illness, he survived COVID-19 and was extubated after 13 days.

## CONCLUSION

This case report adds to the current literature as there are no other current reports of meningitis-like presentation of COVID-19 or herpes zoster. We would also like to draw attention to the patient’s waxing and waning symptoms. As this virus spreads, patients are more likely to present with COVID-19. In addition, clinical studies from South Korea are concerning for an asymptomatic population of ~30% of all virus carriers. The asymptomatic population as well as the varied presentations make a compelling argument for placing every ED patient under precautions on arrival. When and if widespread accurate testing is available, it would be prudent to be able to test all patients, especially those admitted to the hospital in order to decrease healthcare-related exposures. This paradigm would represent a shift in medicine as pre-test probability and testing based on symptoms is critical in our evaluation of patients. However, in a pandemic that is widespread and variable, a different tactic may be indicated.

## Figures and Tables

**Table 1:** Clinical course from onset of atypical symptoms until discharge of patient with presumptive coronavirus 19.

Timeline	Event
Day 1	Symptoms including abdominal pain, constipation start. Progresses to include headache, fever.
Day 3	Initial presentation with headache, fever, abdominal pain. Discharged from emergency department.
Day 9	Re-presents with headache, fever, and concern for meningitis. Undergoes lumbar puncture and admitted to general medicine for meningitis-like presentation.
Day 11	Respiratory decompensation with evidence of bilateral multilobar pneumonia on chest radiograph and computed tomography of the chest.
Day 24	Extubated to nasal cannula in the intensive care unit, suffered from encephalopathy.
Day 27	Patient stable and transferred to the floor.
Day 32	Patient discharged home.

**Table 2:** Literature review of coronavirus 19 symptom presentation.

	Jiaojiao et al^1^ (n =54)	Easom et al^2^ (n=68)	Huang et al^3^ (n=41)	Wang et al^4^ (n=138)	Deng et al^5^ (n = 225)	Guan et al^6^ Meta-analysis (n=1099)
Fever	66.7%	40%	98%	98.6%	80.5%	43.8% (initial) 88.6% (total)
Cough	31.5%	78%	76%	59.4%	37.7%	67.8%
Sputum production	5.6%	28%	-	26.8%	21.7%	33.7%
Fatigue	6.7%	-	44% includes myalgia	69.6%	25.3% includes myalgia	38.1%
Dyspnea	9.3%	25%	5%	31.2%	44%	18.7%
Chest pain/palpitations	7.4%	13%	-	-	10.6%	-
Myalgia	5.6%	16%	See fatigue	34.8%	See fatigue	14.9%
Anorexia	5.6%	-	-	39.9%	-	-
Diarrhea	-	13%	3%	10.1%	14.6%	3.8%
Headache	-	4%	8%	6.5%	5.7%	13.6%
Sore throat	-	1.9%	-	17.4%	-	-
Hemoptysis	-	-	5%	-	3.1%	0.9%
Nasal congestion	1.9%	29%	-	-	-	4.8%
